# Emerging resistance in staphylococci following long-term dalbavancin treatment for prosthetic joint infections

**DOI:** 10.1093/jac/dkag218

**Published:** 2026-06-29

**Authors:** Ronja Hugg, Marc Stegger, Bo Söderquist

**Affiliations:** School of Medical Sciences, Faculty of Medicine and Health, Örebro University, Örebro, Sweden; Department of Orthopedics, Faculty of Medicine and Health, Örebro University, Örebro, Sweden; School of Medical Sciences, Faculty of Medicine and Health, Örebro University, Örebro, Sweden; Department of Sequencing and Bioinformatics, Statens Serum Institut, Copenhagen, Denmark; Antimicrobial Resistance and Infectious Diseases Laboratory, Harry Butler Institute, Murdoch University, Perth, Australia; School of Medical Sciences, Faculty of Medicine and Health, Örebro University, Örebro, Sweden; Department of Orthopedics, Faculty of Medicine and Health, Örebro University, Örebro, Sweden

## Abstract

**Background and objectives:**

Dalbavancin, a lipoglycopeptide with a half-life of 150–200 h, is a promising treatment option for prosthetic joint infections (PJIs) and other orthopaedic implant-associated infections (IAIs) caused by multidrug-resistant staphylococci. However, *in vitro* studies have shown that when exposed to low concentrations of dalbavancin, staphylococcal strains resistant to dalbavancin can emerge, potentially affecting its effectiveness in cases of recurrent infection. To investigate whether dalbavancin-resistant staphylococci emerge *in vivo* following long-term dalbavancin treatment for PJIs or orthopaedic IAIs.

**Patients and methods:**

Nineteen patients who had received long-term dalbavancin treatment (≥12 weeks) following PJI or orthopaedic IAI and 25 control patients scheduled for elective prosthetic joint surgery were sampled from the nares and perineum. Each sample was subcultured on Mueller–Hinton II agar plates containing various concentrations of dalbavancin (0.0, 0.125, 0.5, and 2.0 mg/L). The growth of staphylococcal colonies was analysed using MALDI-TOF, and the MIC values of dalbavancin were determined using the gradient test method.

**Results:**

Among dalbavancin-treated patients, 4 out of 19 displayed staphylococcal species resistant to dalbavancin (MIC value >0.25 mg/L according to EUCAST breakpoint tables). These four were all *Staphylococcus epidermidis* isolates, three from the nares and one from the perineum, and displayed MIC values of 0.38, 0.38, 0.5, and 0.75 mg/L. No resistant staphylococci were detected in the samples from the control group (*P* = 0.029, Fisher's exact test).

**Conclusions:**

The present study demonstrated the emergence of dalbavancin-resistant staphylococci following long-term treatment.

## Introduction

Dalbavancin is an intravenous lipoglycopeptide antibiotic approved by the EMA for the treatment of acute bacterial skin and skin-structure infections. Furthermore, due to its pharmacodynamic properties, dalbavancin has been used ‘off-label’ for the long-term treatment of bone and joint infections, including infections occurring after arthroplasty.^1^ Prosthetic joint infection (PJI) is an uncommon but feared complication that results in increased morbidity and mortality.^2^ The management of PJIs includes both surgical interventions, with debridement, antibiotics, and implant retention (DAIR) or a one- or two-stage exchange surgery, and a prolonged antibiotic regimen, with initial intravenous treatment for approximately 1–2 weeks followed by oral treatment for varying lengths of time. The total treatment period usually lasts 12 weeks.^3^ The microbial aetiology of PJIs is dominated by staphylococci, which mainly represent commensal skin bacteria. Of these, CoNS including *Staphylococcus epidermidis*, account for at least one-third of PJIs.^4,5^ In addition, CoNS often display MDR and have the capacity to produce biofilm in implant-associated infections (IAI).^6^

There are only a few available antibiotic options for treatment of PJI or other orthopaedic IAI involving MDR *S. epidermidis*; one of these is dalbavancin, which has a broad spectrum of activity against Gram-positive pathogens including MDR and methicillin-resistant staphylococci. Furthermore, *in vitro* data have shown that dalbavancin also exerts activity in biofilm, including those produced by methicillin-resistant *S. epidermidis*, at concentrations that are easily achieved *in vivo*.^7,8^ The pharmacokinetic properties of dalbavancin include an exceptionally long half-life of 204 h and a terminal elimination half-life that extends to 346 h.^9^ This allows for an extended dosing interval, which contributes to reduced inpatient treatment time due to the possibility of discharging patients early, thereby lowering healthcare costs and providing dalbavancin as a treatment option for PJIs or other IAIs.

However, due to prolonged terminal elimination, measurable concentrations of dalbavancin have been detected in the serum for more than 3 months after the last dose.^10^ Theoretically, this also indicate that subtherapeutic concentrations of dalbavancin are present for a longer period, which may lead to the emergence and selection of resistance in staphylococcal clones. Dalbavancin is primarily eliminated by the kidneys; however, up to 20% is excreted via the bile into the intestine and may therefore affect the normal flora of the gut, where staphylococci are also found. This may contribute to the selection of resistant clones in the intestine.^9,11^  *In vitro* studies have shown that dalbavancin resistance emerges when staphylococci are exposed to low concentrations of the drug.^12^ The underlying mechanism is not fully understood, but may include the selection of pre-existing heteroresistant subpopulations, epigenetic changes, and spontaneous mutations in regions involved in cell wall synthesis.^12^ Analysis of genomic data from clinical isolates indicated that alterations in the *walK* gene under antibiotic pressure result in, among other effects, an increase in cell wall thickness, thereby reducing susceptibility.^13^

The potential development of resistance can impact the effectiveness of the antibiotic in cases of recurrent infection with the same aetiological agent, thus affecting the indication for further antibiotic treatment. Repeated treatment with dalbavancin may not be a safe alternative from the perspective of resistance if the patient is at risk of colonization by resistant bacterial strains. The question of whether dalbavancin is safe for repeated use has been raised, but as yet remains unanswered.

The aim of this study was to investigate whether dalbavancin-resistant strains of staphylococci, primarily CoNS, were present in the normal flora of the skin and mucous membranes following long-term treatment with dalbavancin due to PJIs or other orthopaedic IAIs.

## Materials and methods

### Patient recruitment and collection of samples

Participants in the treatment group were recruited from a cohort of patients (*n* = 29) who had received long-term treatment with dalbavancin at the Department of Orthopedics, Region Örebro County, Sweden, due to orthopaedic infections (mainly PJIs) from 2022 to 2024. After a written informed consent form was signed, the patients were asked to perform sampling. Nineteen patients agreed to participate and returned their samples. As a control group, 25 patients scheduled for elective primary prosthetic joint surgery were included in the study; all of these provided their written informed consent prior to sample collection.

The participants had to be 18 years or older to be included in any of the study groups. In the treatment group, patients were eligible if they had been treated with at least five doses of dalbavancin, corresponding to at least 12 weeks of treatment, in the last 3 years due to a PJI or an orthopaedic IAI. The characteristics of the participants are presented in Table 1. For the dalbavancin-treated patients, initial or empirical treatment was administered in eight cases with daptomycin and two cases with vancomycin before dalbavancin was administered. The median interval between treatment discontinuation and sampling was 59 weeks (range 6.5–98 weeks). In addition, one patient was receiving ongoing prolonged suppressive treatment with dalbavancin 1500 mg every 6 weeks. Samples were collected through self-sampling from the nares and perineum using eSwab (Copan Italia S.p.A., Brescia, Italy) according to the written instructions. The samples were sent to the Department of Laboratory Medicine, Clinical Microbiology, Örebro University Hospital, Sweden, for further analysis.

**Table 1.** Baseline characteristics of participants

Characteristics	Control patients (*n* = 25)	Dalbavancin-treated patients (*n* = 19)
Sex, *n* (%)		
Male	15 (60%)	16 (84%)
Female	10 (40%)	3 (16%)
Age in years, mean (range)	69 (42–87)	69 (53–88)
Site of joint replacement surgery, *n* (%)		
Hip	18 (72%)	14 (74%)
Knee	7 (28%)	3 (16%)
Other^a^	—	2 (10%)^b^

^a^Other orthopaedic implant-associated surgery.

^b^Ankle prosthesis (*n* = 1) and spinal surgery with osteosynthesis material (*n* = 1).

### Growth of staphylococci and determination of dalbavancin resistance

Each sample was subcultured on Mueller–Hinton II agar plates (Oxoid, Basingstoke, Hampshire, England) containing different concentrations of dalbavancin (0.0, 0.125, 0.5, and 2.0 mg/L) in order to identify resistant bacterial subpopulations. A concentration of 0.125 mg/L was chosen based on the EUCAST breakpoint for dalbavancin against *Staphylococcus aureus* at the onset of the study (EUCAST v_14.0, 2024), and concentrations of 0.5 and 2.0 mg/L were included to represent two successive 2-fold dilution steps. The plates were incubated in air at 36°C for 48 h. Colonies growing on dalbavancin-containing plates were analysed using MALDI-TOF MS (Microflex LT and Biotyper 3.1, Bruker Daltonics, Bremen, Germany) to confirm the species. A gradient test was performed to determine the antibiotic susceptibility and MIC values for dalbavancin. A 0.5 McFarland suspension of bacteria in 0.85% NaCl was dispensed on Mueller–Hinton II agar plates, and gradient strips (MIC Test Strips, Liofilchem, Roseto degli Abruzzi, Italy) were placed on the plates and incubated for 16 h at 35°C. The MIC values were manually read, and susceptibility was interpreted according to the latest EUCAST clinical breakpoint (v_15.0, 2025).

The identified resistant *S. epidermidis* isolates were stored at −80°C in a preservation medium consisting of trypticase soy broth supplemented with 0.3% yeast extract (BD Diagnostic Systems, Sparks, MD, USA) and 29% horse serum (Håtunalab AB, Håtuna, Sweden).

### Genome sequencing and analyses

Genomic DNA was extracted from four staphylococcal isolates using a MagNA Pure 96 automated extraction platform (Roche, Basel, Switzerland) with the DNA and Viral NA Small Volume kit (Roche, Basel, Switzerland). Sequencing libraries were prepared using a custom-modified version of the DNA prep kit (Illumina, San Diego, CA, USA) based on the Hackflex protocol.^14^ The quality of the final sequencing libraries was assessed by measuring the concentration on a Qubit 3.0 fluorometer prior to sequencing on a NextSeq 1000 (Illumina) using a P1 300-cycle reagent kit generating 2×150 bp paired-end reads. Quality control and typing of the raw fastq sequences was performed using Bifrost (https://github.com/ssi-dk/bifrost). Genome analysis was performed using DotMatics Geneious Prime for investigations of resistance-associated mutation in the *walK* gene.

### Statistics

Because of the small sample size and the presence of zero events in the control group, Fisher's exact test was used to compare the two groups (controls and dalbavancin-treated patients) in terms of the prevalence of resistant staphylococci and the growth of any staphylococci in samples from the normal flora. The statistical analysis was conducted using IBM SPSS Statistics (IBM Corp., Armonk, NY, USA). A *P* value <0.05 was considered statistically significant.

### Ethics

The study was approved by the Swedish Ethical Review Authority in Uppsala, Sweden (reference number: 2025-00565-01). All participants in both groups provided their written informed consent before participating.

## Results

### Recruitment of study participants

We included 44 patients in the study, comprising 19 dalbavancin-treated patients (17 following PJI in either the hip or knee and 2 following an orthopaedic IAI) and 25 control patients who were scheduled for elective joint replacement surgery. The initial recruitment goal was 50 patients, with 25 in each group. We asked 29 dalbavancin-treated patients to participate, and 19 agreed to return samples.

### Growth of staphylococci

After 48 h of incubation, agar plates containing varying concentrations of dalbavancin were examined for the presence of staphylococcal colonies. Growth of staphylococci on any of the four agar plates was observed in all 19 nares samples obtained from the dalbavancin-treated patients and in 23 out of 25 nares samples from the control group (Table 2). The corresponding figures for the perineal samples were 7/19 samples from the dalbavancin-treated patients and 21/25 samples from the controls. However, the antibiotic concentration at which staphylococcal growth was inhibited differed between individual patients. Details regarding the growth patterns in relation to the dalbavancin concentration are given in Appendices 1 and 2 (available as Supplementary data at *JAC* Online). Additionally, the number of staphylococci growing on plates with varying concentrations of dalbavancin differed between the control and dalbavancin treatment groups (Table 3). Isolates from treated patients were more likely to display growth of >40 colonies in the nares, regardless of the antibiotic concentration in the agar plates, compared with the control group. In contrast, the number of staphylococci in the perineal samples from the treated patients was lower than that in the control patients.

**Table 2.** Growth of staphylococcal colonies in samples from the nares and perineum of 25 control patients and 19 dalbavancin-treated patients, after 48 h incubation on agar plates

	Growth of staphylococcal colonies		*P* value*
	Control patients (*n* = 25)	Dalbavancin-treated patients (*n* = 19)	
Nares	23 (92%)	18 (95%)	1.0
Perineum	21 (84%)	7 (37%)	0.002

Samples were incubated on agar plates containing four different concentrations of dalbavancin (0, 0.125, 0.5, and 2.0 mg/L). Results are reported as number of patients with growth of staphylococci on any of the four agar plates.

^*^Growth of staphylococci in the normal flora in patients in the dalbavancin-treatment group compared with control group using Fischer's exact test. *P* value <0.05 was considered statistically significant.

**Table 3.** Distribution of number of staphylococcal colonies identified in nares and perineal samples from 25 control patients and 19 dalbavancin-treated patients after 48 h incubation on agar plates containing different concentrations of dalbavancin

	No colonies		<20 Colonies		20–40 Colonies		>40 Colonies	
	Control	Treated	Control	Treated	Control	Treated	Control	Treated
0 mg/L								
Nares	2	1	5	4	7	0	11	14
Perineum	4	11	1	2	4	1	16	4
0.125 mg/L								
Nares	3	1	14	4	4	1	4	13
Perineum	4	12	5	1	11	1	6	4
0.5 mg/L								
Nares	6	3	18	8	1	3	0	5
Perineum	6	13	16	4	3	0	0	1
2.0 mg/L								
Nares	10	5	15	8	0	2	0	4
Perineum	11	14	14	3	0	1	0	0

### Resistance and MIC value

**Figure 1.**
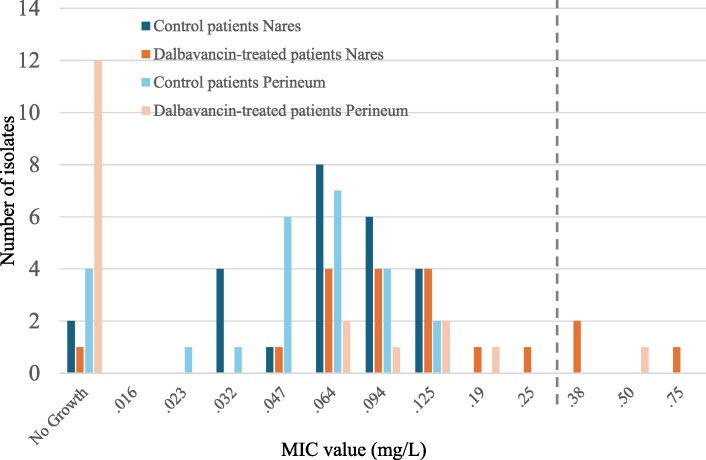
Distribution of the highest measured MIC value for any staphylococcal colony in nares and perineal samples from 25 control patients and 19 dalbavancin-treated patients. Values were determined with gradient strips for dalbavancin. The grey dashed line indicates the EUCAST breakpoint for dalbavancin against staphylococcal strains (>0.25 mg/L; EUCAST breakpoint tables v_15.0).

Four of the 19 patients with long-term dalbavancin treatment were colonized with a staphylococcal strain that displayed resistance to dalbavancin (i.e. a MIC value of  > 0.25 mg/L according to the latest EUCAST breakpoint tables; v_15.0, 2025) at the time of the sampling. These four were all *S. epidermidis* isolates, three from the nares and one from the perineum, and showed MIC values of 0.38, 0.38, and 0.75 for the samples from the nares and 0.5 mg/L for the sample from the perineum. The time between treatment discontinuation and sampling was 6.5, 61, and 98 weeks, respectively, and the last patient was still receiving suppressive treatment with dalbavancin every 6 weeks. The prevalence of dalbavancin-resistant *S. epidermidis* was higher in the treated group than in the control group (4/19 (21%) versus 0/25 (0%), *P* = 0.029). No staphylococci with MIC values >0.25 mg/L were detected in the samples from the control patients. The distribution of the highest measured MIC values for isolates from dalbavancin-treated and control patients is presented in Figure 1.

The identified resistant *S. epidermidis* isolates stored at −80°C were thawed and re-tested twice using the gradient test as described above. In addition, BMD according to ISO 20776-1 was performed at EUCAST Development Laboratory, Clinical Microbiology, Växjö Hospital, Växjö, Sweden (Table 4).

**Table 4.** MIC determination of dalbavancin-resistant *Staphylococcus epidermidis* isolated from nares (N) and perineal (P) samples from dalbavancin-treated patients

Isolate ID	Dalbavancin (mg/L)	Dalbavancin (mg/L)	Dalbavancin (mg/L)	Vancomycin (mg/L)
	Gradient test	Gradient test	BMD	BMD
	Study	Re-test		
D1N	0.38	0.75	1	4
D2N	0.38	1.5	1	8
D11P	0.5	0.38	0.25	4
D16N	0.75	0.75	1	8

### Genome sequencing

Genomic analysis of *walK* gene did not reveal any amino acid changes in the WalK(V500F) and WalK(Q371del/P415L) positions. Of the four resistant isolates, three belonged to ST 2, and one to ST283. This latter type is not associated to any major hospital-adapted lineages.

## Discussion

In this observational *in vivo* study, using a breakpoint of MIC >0.25 mg/L we found dalbavancin-resistant staphylococci, all *S. epidermidis*, in the normal flora of more than 20% of patients who had undergone long-term dalbavancin treatment, compared with none in the control group.

Dalbavancin is a promising treatment option for the management of PJIs due to its favourable tissue penetration, potent activity against MDR staphylococci, and suitability for outpatient administration. Nevertheless, the findings of this *in vivo* study raise important clinically relevant concerns. The long half-life of dalbavancin and prolonged terminal elimination with subtherapeutic concentrations have been reported in several *in vitro* studies as risk factors for the development of resistant staphylococci by extending the mutant selection window, suggesting that staphylococci with reduced susceptibility can emerge when exposed to dalbavancin.^12,15–17^ In five available *in vivo* case reports, resistance to dalbavancin was demonstrated following dalbavancin treatment for PJIs or infective endocarditis.^12,13,18–20^

This observed resistance could potentially compromise the clinical utility of dalbavancin. Patients who undergo arthroplasty surgery of another joint in the future may still carry resistant clones on their skin or mucous membranes. Furthermore, some PJI patients treated with DAIR followed by long-term antibiotic treatment may need to undergo a subsequent one- or two-stage exchange surgery due to failure. If this subsequent surgery occurs within months after dalbavancin treatment, these patients may be at an increased risk of new infections with dalbavancin-resistant staphylococci, due to the emergence of resistance. To overcome this problem, ensuring adequate serum concentrations is important to reduce the risk of subtherapeutic concentrations during the treatment period.^15^

In the current EUCAST breakpoint table (v_15.0, valid from 2025-01-01), the breakpoint for staphylococci has been revised, and an MIC value >0.25 mg/L is now regarded as resistant. The previous EUCAST table (v_14.0) had an MIC breakpoint value of 0.125 mg/L and was based exclusively on *S. aureus* isolates, whereas the updated version encompasses other staphylococcal species as well. This change in the MIC breakpoint value could indicate uncertainty about how to define the exact breakpoint, indicating that further research is needed, especially to explore the wild-type distribution.^21^

In this study, we observed a lower level of staphylococcal colonization in the perineal samples among dalbavancin-treated patients compared with controls (*P* = 0.002). This indicates that perineal samples, which in part represent the normal flora of the perineum but also may reflect the gut, can be affected by long-term dalbavancin treatment. Antibiotic treatment can harm normal flora for a period after treatment, and may disrupt the composition of the gut microbiome by selecting harmful bacteria.^22^ The present small study cannot confirm whether this happens following long-term dalbavancin treatment, but the results may indicate an alteration of the normal flora, at least with a reduction in staphylococcal colonization.

We also observed that staphylococci appeared to grow more heavily in the nasal flora of patients who had undergone long-term dalbavancin treatment than in the nasal flora of control patients, which may indicate a change in the normal flora of the skin. The clinical significance of this finding remains to be investigated, and requires further studies. It may also be of interest to assess the duration of any potential alterations to the normal skin flora following treatment. In the present study, sampling was conducted at various time points post-treatment, with the longest interval being 3 years after the completion of therapy. To accurately interpret the potential changes in the normal flora, and to be able to determine persistence over time, it would be of value in future studies to account for the temporal relationship between treatment and sampling.

An unexpected finding in this study was that there was a relatively high number of staphylococcal colonies growing on agar plates containing dalbavancin concentrations above the established breakpoint MIC value; however, when subsequent susceptibility testing using the gradient test was performed, only a few of the isolates displayed a resistant phenotype. The effective antibiotic concentration on the agar surface may have been lower than intended, or staphylococci may have displayed transient traits that allowed them to grow despite the presence of dalbavancin. Nevertheless, this is unlikely to have influenced the overall outcome, as resistant strains would likely have been identified regardless of the extent of surface growth.

No mutations in the *walK* gene could be demonstrated for the four isolated strains displaying MIC values >0.25 mg/L; however, other unexplored genetic mechanisms may be present.

A limitation of this study is the low number of recruited patients, which was due to the short study period and the low number of dalbavancin-treated patients at the study site. In addition, the response rate was low in the treatment group. Despite follow-up reminders, 10 patients did not return any samples. This may, in part, be attributed to the advanced age and comorbidities of the study population, which could have made it more challenging for patients to independently complete and return the samples. Another limitation is that the samples were collected from only two sites, the nares and the perineum, to reflect the normal flora of the skin and intestine. Sampling was also performed through self-sampling according to written instructions, which limited the ability to ensure proper sampling technique. However, all submitted samples yielded bacterial growth when cultured on agar plates. Although not all samples contained staphylococcal strains, the presence of bacterial growth indicated that sampling was successful. Self-sampling was chosen to facilitate study participation by eliminating the need for hospital visits; nevertheless, future studies may benefit from the sequential collection of samples from various sites during scheduled follow-up visits to ensure sampling quality and consistency.

The present investigation indicates that further, larger prospective studies should be performed to confirm the results of this study. It would be of interest to investigate how long dalbavancin-resistant strains persist after the last dose, because previous findings imply that resistance to other antibiotic agents disappears weeks after stopping antibiotic treatment due to the contributing resistance mechanism and fitness cost.^23^ Genetic changes, such as mutations, would contribute to more persistent resistance, whereas epigenetic changes could theoretically cause more transient resistance.^12^ In the present study, sampling was conducted at varying time points post-treatment due to the retrospective observational study design; therefore, we did not consider how long ago the treatment group received their last dose of dalbavancin. Theoretically, resistance could have been present earlier in patients who did not show resistant staphylococci in the normal flora, thus making the result even more significant. To investigate the persistence of resistant strains within the normal flora, a structured longitudinal sampling protocol with sampling at multiple time points is required, accounting for the temporal relationship between the last dose and sample collection.

### Conclusions

The present study demonstrated the emergence of dalbavancin-resistant staphylococci *in vivo* in the normal flora following long-term dalbavancin treatment for PJIs. Nearly, one-quarter of the treated patients carried dalbavancin-resistant staphylococci, compared with none in the control group. These findings do not challenge the established clinical efficacy of dalbavancin but highlight the need for caution regarding prolonged or repeated exposure and future reuse in the same patient.

## Supplementary Material

dkag218_Supplementary_Data

## Data Availability

The datasets generated and analysed during the current study are available from the European Nucleotide Archive under BioProject accession number PRJEB113463.

## Supplementary data

Appendices 1 and 2 are available as Supplementary data at *JAC* Online.
