# Supplementary material for: Emerging resistance in staphylococci following long-term dalbavancin treatment for prosthetic joint infections
Source: J Antimicrob Chemother. 2026 Jun 29;81(7):dkag218. doi: 10.1093/jac/dkag218 (PMC13312031; doi:10.1093/jac/dkag218)
Supplement: dkag218_Supplementary_Data [file dkag218_supplementary_data.docx]

Supplementary Material

| Appendix 1. Growth or no growth of staphylococcal colonies after 48 h incubation on agar plates containing four different concentrations of dalbavancin, reported for samples from nares and perineum from each control patient (n=25). | | | | |
| --- | --- | --- | --- | --- |
|  | **0 mg/L** | **0.125 mg/L** | **0.5 mg/L** | **2.0 mg/L** |
| C1  Nares  Perineum | x  - | x  - | x  - | -  - |
| C2  Nares  Perineum | x  x | x  x | x  - | x  - |
| C3  Nares  Perineum | x  x | x  x | x  x | -  x |
| C4  Nares  Perineum | x  - | x  - | x  - | x  - |
| C5  Nares  Perineum | x  - | x  - | x  - | x  - |
| C6  Nares  Perineum | x  x | x  x | -  x | -  - |
| C7  Nares  Perineum | x  x | x  x | x  x | x  x |
| C8  Nares  Perineum | x  x | x  x | x  x | x  x |
| C9  Nares  Perineum | x  x | x  x | -  x | -  - |
| C10  Nares  Perineum | x  x | x  x | x  x | x  x |
| C11  Nares  Perineum | x  x | x  x | x  x | x  x |
| C12  Nares  Perineum | x  x | x  x | x  x | x  x |
| C13  Nares  Perineum | x  x | x  x | -  x | -  - |
| C14  Nares  Perineum | x  x | x  x | x  x | x  - |
| C15  Nares  Perineum | x  x | x  x | x  x | x  x |
| C16  Nares  Perineum | x  x | x  x | x  x | -  x |
| C17  Nares  Perineum | x  x | x  x | x  - | x  - |
| C18  Nares  Perineum | x  x | x  x | x  x | x  x |
| C19  Nares  Perineum | x  x | x  x | x  x | x  x |
| C20  Nares  Perineum | x  x | x  x | x  x | x  x |
| C21  Nares  Perineum | -  x | -  x | -  x | -  x |
| C22  Nares  Perineum | x  - | x  - | x  - | -  - |
| C23  Nares  Perineum | x  x | x  x | x  x | x  x |
| C24  Nares  Perineum | x  x | x  x | x  x | -  - |
| C25  Nares  Perineum | -  x | -  x | -  x | -  x |
| x = growth of staphylococcal colonies, - = no growth of staphylococcal colonies | | | | |

| Appendix 2. Growth or no growth of staphylococcal colonies after 48 h incubation on agar plates containing four different concentrations of dalbavancin, reported for samples from nares and perineum from each dalbavancin-treated patient (n=17). | | | | |
| --- | --- | --- | --- | --- |
|  | **0 mg/L** | **0.125 mg/L** | **0.5 mg/L** | **2.0 mg/L** |
| D1  Nares  Perineum | x  x | x  x | x  x | x  x |
| D2  Nares  Perineum | x  - | x  - | x  - | x  - |
| D3  Nares  Perineum | x  - | x  - | x  - | -  - |
| D4  Nares  Perineum | x  - | x  - | x  - | x  - |
| D5  Nares  Perineum | x  - | x  - | x  - | x  - |
| D6  Nares  Perineum | x  x | x  x | x  x | x  x |
| D7  Nares  Perineum | x  x | x  x | x  - | x  - |
| D8  Nares  Perineum | x  - | x  - | x  - | x  - |
| D9  Nares  Perineum | x  - | x  - | x  - | x  - |
| D10  Nares  Perineum | x  - | x  - | x  - | x  - |
| D11  Nares  Perineum | x  - | x  - | x  - | x  - |
| D12  Nares  Perineum | x  - | x  - | x  - | x  - |
| D13  Nares  Perineum | x  x | x  x | -  x | -  x |
| D14  Nares  Perineum | x  x | x  - | -  - | -  - |
| D15  Nares  Perineum | x  - | x  - | x  - | x  - |
| D16  Nares  Perineum | x  x | x  x | x  x | -  x |
| D17  Nares  Perineum | x  x | x  x | x  x | x  x |
| x = growth of staphylococcal colonies, - = no growth of staphylococcal colonies | | | | |
